# The Effects of Exercise on Cognitive Function in Older Adults With Different Types of Dementia: A Meta-Analysis

**DOI:** 10.1093/geroni/igab046.2893

**Published:** 2021-12-17

**Authors:** Guilherme Balbim, Ryan Falck, Cindy Barha, Jennifer Davis, Samantha Starkey, Alexis Bullock, Teresa Liu-Ambrose

**Affiliations:** 1 University of British Columbia, Vancouver, British Columbia, Canada; 2 University of British Columbia-Okanagan, Kelowna, British Columbia, Canada; 3 the University of British Columbia, Vancouver, British Columbia, Canada

## Abstract

Combating dementia is a public health priority, and exercise training is one promising strategy for dementia prevention. However, its efficacy in promoting cognitive outcomes in different types of dementia remains unknown. We conducted a systematic review (N = 27) and meta-analysis (N = 24) of randomized controlled trials with cognitive function as a primary or secondary outcome. We aimed to assess the effect of exercise interventions on the cognitive function of older adults (>60 years) diagnosed with different types of dementia. We synthesized data from 2,441 older adults with dementia. Eleven trials included older adults with multiple types of dementia, eight with Alzheimer's disease, six with unspecified types of dementia, and two with vascular cognitive impairment. We performed random-effects models using robust variance estimation (RVE) and tested potential moderators using the approximate Hotelling-Zhang test (HTZ). Results suggest a small effect of exercise on cognitive function for all-cause dementia (g = 0.18; 95% CI: 0.04, 0.33; p = 0.016); however, the effects did not differ by type of dementia. Moderation analyses showed that trials that did not specify participants' severity of dementia, applied individual-level randomization, and had higher intervention adherence demonstrated larger exercise effects on cognitive function for all-cause dementia. We conclude that exercise promotes small improvements in the cognitive function of older adults with all-cause dementia. More research including different types of dementia is needed if we hope to determine the precise effects of exercise for each type of dementia.

